# β-mannanase supplemented in diets saved 85 to 100 kcal of metabolizable energy/kg, supporting growth performance and improving nutrient digestibility in grower pigs

**DOI:** 10.1038/s41598-023-38776-5

**Published:** 2023-08-02

**Authors:** Jansller Luiz Genova, Liliana Bury de Azevedo, Paulo Evaristo Rupolo, Flávia Beatriz Carvalho Cordeiro, Hellen Lazarino Oliveira Vilela, Pedro Silva Careli, Damares de Castro Fidelis Toledo, Silvana Teixeira Carvalho, Marcos Kipper, Luciana Navajas Rennó, Juliana Canto Faveri, Paulo Levi de Oliveira Carvalho

**Affiliations:** 1 Animal Science Department, Universidade Federal de Viçosa, Viçosa, 36570900 Brazil; 2 Animal Science Department, Universidade Estadual do Oeste do Paraná, Marechal Cândido Rondon, 85960000 Brazil; 3 Animal Science Department, Universidade Federal da Bahia, Salvador, 40110909 Brazil; 4 Elanco Animal Health Incorporated Company, São Paulo, 04794000 Brazil

**Keywords:** Animal physiology, Multienzyme complexes, Cytokines, Metabolism, Metagenomics

## Abstract

The effects of β-mannanase supplementation in metabolizable energy (ME)-reduced diets containing xylanase-phytase were investigated on growth performance, fecal score, ultra-sounded backfat thickness and loin depth, blood profile, apparent total tract digestibility (ATTD), digesta passage rate, and fecal microbiome in grower pigs (n = 40, 26.09 ± 0.96 kg) randomly assigned within 4 treatments: a control diet containing isolated phytase and xylanase valued at 40 kcal of ME/kg (CD0), CD0 + β-mannanase (0.3 g/kg valued at 30 kcal of ME/kg) (CD70), CD0 + β-mannanase (0.3 g/kg valued at 45 kcal of ME/kg) (CD85), and CD0 + β-mannanase (0.3 g/kg valued at 60 kcal of ME/kg) (CD100). Growth performance was not affected in pigs fed ME-reduced diets containing β-mannanase. Pigs with CD100 had lower serum IL-1β concentration, and higher IL-10 was observed in pigs on CD0 than those fed β-mannanase. Coefficients of ATTD, and ATTD of DM and CP were higher in animals fed CD85 or CD100. Pigs with CD85 had higher alpha diversity richness but lower Firmicutes:Bacteroidota ratio. Acidaminococcaceae and Ruminococcaceae were more abundant in pigs fed CD0, but lower for *Christensenellaceae NSJ-63* and *NSJ-63 sp014384805*. Pigs in CD85 showed higher Bacteroidaceae and *Prevotella* abundance, and lower for Streptococcaceae and *Streptococcus*. In conclusion, supplementation of β-mannanase in diets containing xylanase-phytase saved 85 to 100 kcal of ME/kg by supporting growth performance and improving nutrient digestibility in grower pigs.

## Introduction

Corn and soybean meal are the most widely used plant-based ingredients globally in pig diet formulations, but they contain 6% to 17% non-starch polysaccharides that are indigestible by monogastrics^1^. In addition, antinutritional factors such as trypsin inhibitors, antigenic factors, phytates, β-mannans^2,3^, and xylans^4^ compromise nutrient usage. Consequently, the use of exogenous enzymes in diets has the role of reducing the negative effects of antinutritional factors, promoting greater digestion and assimilation of nutrients, and improving health and growth performance of pig and poultry^5^.

Although increasing attention has been given to the use of exogenous enzymes in pig nutrition, few studies have considered the combination of β-mannanase-xylanase-phytase enzymes. It is still unclear about the "extra phosphorus" effects of phytase in providing additional energy^6^, and the favorable action of β-mannanase on the immune response and intestinal microbiome by degrading β-mannans^7,8^. Also, there is little agreement on the association of xylanase-phytase in releasing more phytic acid^9^ and in modulating the growth of pathogenic microorganisms by reducing digesta viscosity in pigs^10^.

Collectively, Petry et al.^11^ found that xylanase supplementation in high-fiber diets for sows promoted increased microbial diversity in the cecal contents and colonic mucosa. In addition, Zhang et al.^12^ evidenced that phytase supplementation in the diets of finisher pigs improved intestinal antioxidant capacity and immune system. There is also a report that β-mannanase supplementation reduced post-weaning diarrhea in young pigs^3^. A reduction in backfat thickness and higher blood glucose concentration was observed in pigs fed diets supplemented with β-mannanase-xylanase^13^. However, the effects of β-mannanase supplemented in ME-reduced diets containing xylanase-phytase have not been addressed so far on blood immune response and intestinal ecosystem level in grower pigs.

Here, our hypothesis is that β-mannanase supplementation in ME-reduced diets containing xylanase-phytase promotes beneficial bacterial diversity by increased ATTD and improves immune response by saving energy expenditure and consequently promotes health in pigs. Therefore, a study was conducted to assess the supplementation of β-mannanase in ME-reduced diets containing xylanase-phytase and its effects on growth performance, fecal score, ultra-sounded backfat thickness and loin depth, blood biochemical and immune profile, ATTD, total digesta passage rate and fecal microbiome in grower pigs.

## Results

### Growth performance, fecal score, and ultra-sounded backfat thickness and loin depth

Growth performance, and ultra-sounded backfat thickness and loin depth were not affected in pigs fed ME-reduced diets supplemented with β-mannanase (Table 1). There was a trend (*P* = 0.082) of higher fecal consistency score in grower I pigs fed CD70 compared to CD85 and CD100, but intermediate results in animals with CD0.

**Table 1:** Effects of β-mannanase supplementation in metabolizable energy-reduced diets containing xylanase-phytase on growth performance, fecal score, and ultra-sounded backfat thickness and loin depth of grower pigs (n = 10 per treatment). ^a^IBW: initial body weight, FBW: final body weight, ADFI: average daily feed intake, DWG: daily weight gain, G:F: gain to feed ratio. ^b^A control diet containing isolated phytase and xylanase valued at 40 kcal of ME/kg (CD0), CD0 + β-mannanase (0.3 g/kg valued at 30 kcal of ME/kg) (CD70), CD0 + β-mannanase (0.3 g/kg valued at 45 kcal of ME/kg) (CD85), and CD0 + β-mannanase (0.3 g/kg valued at 60 kcal of ME/kg) (CD100). ^c^SEM: pooled standard error of the mean.

Item^a^	Treatments^b^				SEM^c^	*P*-value
	CD0	CD70	CD85	CD100		
Grower I (day 0 to 25)						
IBW, kg	26.00	26.09	26.11	26.14	0.20	0.474
FBW, kg	51.05	50.10	50.50	49.75	0.40	0.153
ADFI, kg	1.82	1.77	1.77	1.75	0.02	0.653
DWG, kg	1.02	0.95	0.97	0.94	0.01	0.214
G:F, kg:kg	0.55	0.54	0.55	0.53	0.50	0.737
Fecal score	1.40	1.70	1.10	1.10	0.09	0.082
Grower II (day 25 to 42)						
FBW, kg	72.50	69.35	71.20	70.50	0.62	0.153
ADFI, kg	2.68	2.44	2.58	2.58	0.03	0.124
DWG, kg	1.26	1.13	1.21	1.22	0.02	0.154
G:F, kg:kg	0.46	0.46	0.47	0.47	0.63	0.957
Fecal score	1.20	0.90	0.90	1.00	0.06	0.278
Overall period (day 0 to 42)						
ADFI, kg	2.17	2.04	2.10	2.09	0.02	0.335
DWG, kg	1.10	1.02	1.07	1.05	0.01	0.147
G:F, kg:kg	0.50	0.50	0.51	0.50	0.43	0.909
Ultrasound backfat thickness, mm	8.30	8.30	7.50	8.00	0.02	0.464
Ultrasound loin depth, mm	38.10	39.70	38.90	40.10	0.05	0.579

### Blood biochemical and immunological profile

There was no effect of dietary treatment on blood biochemical profile; however, a trend (*P* = 0.057) of higher blood triglyceride concentration was observed in pigs fed CD0 compared to CD85 and CD100, but intermediate results in animals fed CD70 (Table 2).

**Table 2:** Effects of β-mannanase supplementation in metabolizable energy-reduced diets containing xylanase-phytase on blood profile in grower pigs at day 42 (n = 10 per treatment). ^a^A control diet containing isolated phytase and xylanase valued at 40 kcal of ME/kg (CD0), CD0 + β-mannanase (0.3 g/kg valued at 30 kcal of ME/kg) (CD70), CD0 + β-mannanase (0.3 g/kg valued at 45 kcal of ME/kg) (CD85), and CD0 + β-mannanase (0.3 g/kg valued at 60 kcal of ME/kg) (CD100). ^b^SEM: pooled standard error of the mean. Pigs fed CD100 diet had (P < 0.05) lower serum IL-1β concentration compared to animals that received .

Item	Treatments^a^				SEM^b^	*P*-value
	CD0	CD70	CD85	CD100		
Albumin, g/dL	2.52	2.54	2.55	2.46	0.03	0.836
Total cholesterol, mg/dL	87.60	83.64	85.03	89.86	3.39	0.930
Triglycerides mg/dL	43.83	35.19	26.45	27.58	2.58	0.057
Glucose, mg/dL	97.51	97.53	93.10	96.20	1.40	0.663
Urea, mg/dL	23.20	22.90	22.63	24.26	0.74	0.886
Total protein, g/dL	7.12	7.03	6.94	7.01	0.08	0.894
Globulin, g/dL	4.60	4.48	4.39	4.55	0.08	0.854

**Figure 1:**
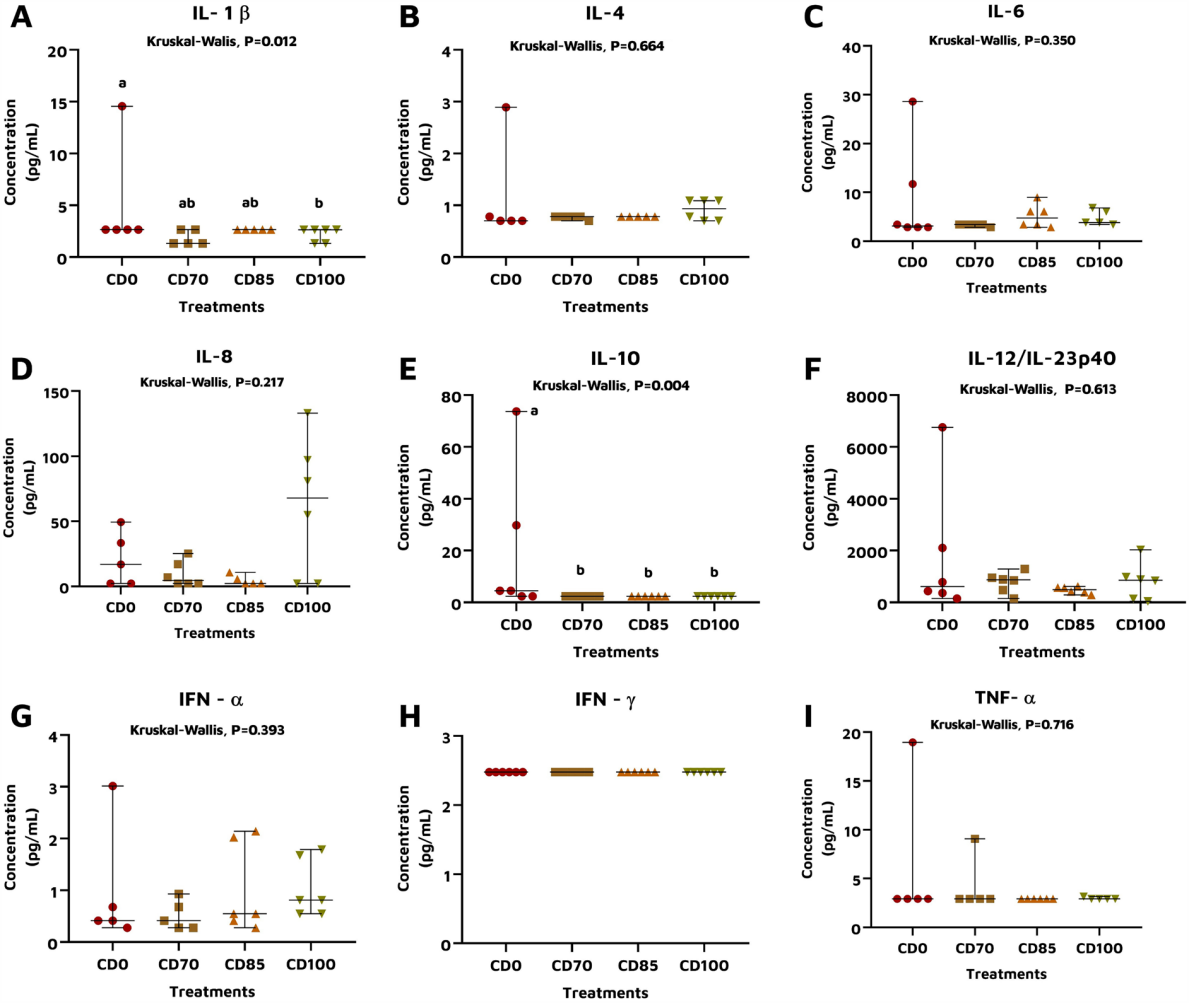
Simultaneous detection of serum concentration (pg/mL) of IL-1β (**A**), IL-4 (**B**), IL-6 (**C**), IL-8 (**D**), IL-10 (**E**), IL-12/23p40 (**F**), IFN-α (**G**), IFN-γ (**H**), and TNF-α (**I**) in grower pigs (n = 8 per treatment) fed 1 of 4 dietary treatments: a control diet containing isolated phytase and xylanase valued at 40 kcal of ME/kg (CD0), CD0 + β-mannanase (0.3 g/kg valued at 30 kcal of ME/kg) (CD70), CD0 + β-mannanase (0.3 g/kg valued at 45 kcal of ME/kg) (CD85), and CD0 + β-mannanase (0.3 g/kg valued at 60 kcal of ME/kg) (CD100). Each of the points represents a sample, the vertical axis represents the range of values, and the horizontal axis refers to the median. Means differed using the Kruskal–Wallis test with Dunn's post hoc (*P* < 0.05).

Pigs fed CD100 diet had (*P* < 0.05) lower serum IL-1β concentration compared to animals that received CD0 diet (Fig. 1A). There was no effect of dietary treatment on the serum concentration of the cytokines IL-4 (Fig. 1B), IL-6 (Fig. 1C), and IL-8 (Fig. 1D). However, a reduction in serum IL-10 concentration was observed (*P* < 0.05) in pigs fed diets supplemented with β-mannanase than animals fed CD0 (Fig. 1E). The interleukin IL-12/23p40 was the cytokine that presented the highest concentration among all cytokines assessed (Fig. 1F). There was no effect of dietary treatment on the serum concentration of the cytokine IFN-α (Fig. 1G). All results were below the detection limit for IFN-γ concentration (Fig. 1H), and no effect of dietary treatment was observed on TNF-α concentration (Fig. 1I).

### Apparent total tract digestibility and total digesta passage rate

Notable results were the highest (*P* < 0.05) ATTD coefficients of DM, and OM in animals fed CD85 or CD100 diets compared to pigs fed CD0 diets, but intermediate results in animals fed CD70 (Table 3). A lower (*P* < 0.05) ATTD coefficient of CP was obtained in pigs fed CD0 or CD70 diets compared to those fed CD85 or CD100. In addition, pigs fed CD85 diets had greater (*P* < 0.05) ATTD coefficient of GE than those fed CD0, but intermediate results were found in animals fed CD70 or CD100. Interestingly, a greater (*P* < 0.05) ATTD of DM and CP was observed in pigs fed CD85 or CD100 diets compared to those fed CD0, and higher ATTD of CP in pigs fed CD85 or CD100 diets than animals fed CD70 diets. However, there was no difference between dietary treatments on total digesta passage rate.

**Table 3:** Effects of β-mannanase supplementation in metabolizable energy-reduced diets containing xylanase-phytase on apparent total tract digestibility at day 42 (as a dry matter basis), and digesta passage rate in grower pigs (n = 10 per treatment). ^a,b^Different from each other according to Tukey's post hoc test (*P* < 0.05). ^A^Apparent total tract digestibility coefficients of dry matter (ADCDM), crude protein (ADCCP), organic matter (ADCOM), gross energy (ADCGE); ATTD of dry matter (DM), ATTD of crude protein (CP), ATTD of organic matter (OM), digestible energy (DE). ^B^A control diet containing isolated phytase and xylanase valued at 40 kcal of ME/kg (CD0), CD0 + β-mannanase (0.3 g/kg valued at 30 kcal of ME/kg) (CD70), CD0 + β-mannanase (0.3 g/kg valued at 45 kcal of ME/kg) (CD85), and CD0 + β-mannanase (0.3 g/kg valued at 60 kcal of ME/kg) (CD100). ^C^SEM: pooled standard error of the mean.

Item^A^	Treatments^B^				SEM^C^	*P*-value
	CD0	CD70	CD85	CD100		
ADCDM, %	80.40^b^	81.97^ab^	82.88^a^	82.97^a^	0.29	0.002
ADCCP, %	77.58^b^	76.98^b^	81.80^a^	81.48^a^	0.57	0.005
ADCOM, %	83.51^b^	85.24^ab^	85.93^a^	85.94^a^	0.28	0.002
ADCGE, %	79.99^b^	81.57^ab^	84.65^a^	83.25^ab^	0.57	0.017
DM, %	80.01^b^	81.55^ab^	82.42^a^	82.51^a^	0.29	0.003
CP, %	14.29^b^	12.96^c^	16.27^a^	15.82^a^	0.22	< 0.001
OM, %	79.63	80.88	80.81	80.82	0.23	0.167
DE, kcal/kg	3730	3823	3898	3798	24.77	0.122
Digesta passage rate on d 25, min	1310	1326	1309	1284	26.25	0.957
Digesta passage rate on d 42, min	1339	1393	1389	1319	28.27	0.754

### Fecal microbiome

**Figure 2:**
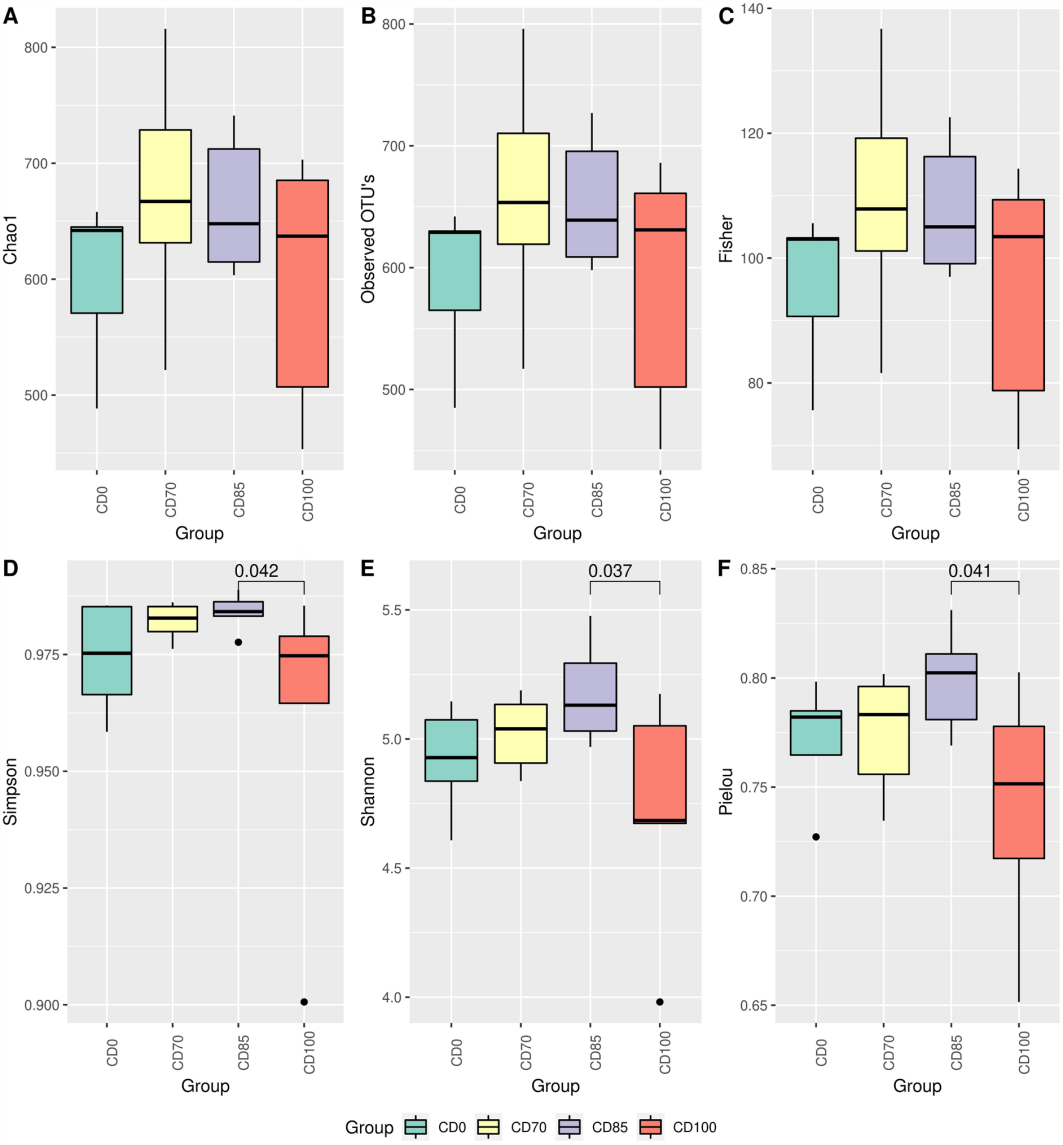
Alpha diversity estimated by parameters Chao1 (**A**), observed OTUs (**B**), Fisher (**C**), Simpson (**D**), Shannon (**E**), and Pielou (**F**) in grower pigs (n = 6 per treatment) fed 1 of 4 dietary treatments: a control diet containing isolated phytase and xylanase valued at 40 kcal of ME/kg (CD0), CD0 + β-mannanase (0.3 g/kg valued at 30 kcal of ME/kg) (CD70), CD0 + β-mannanase (0.3 g/kg valued at 45 kcal of ME/kg) (CD85), and CD0 + β-mannanase (0.3 g/kg valued at 60 kcal of ME/kg) (CD100). Means differed by Wilcoxon test (*P* < 0.05).

**Figure 3:**
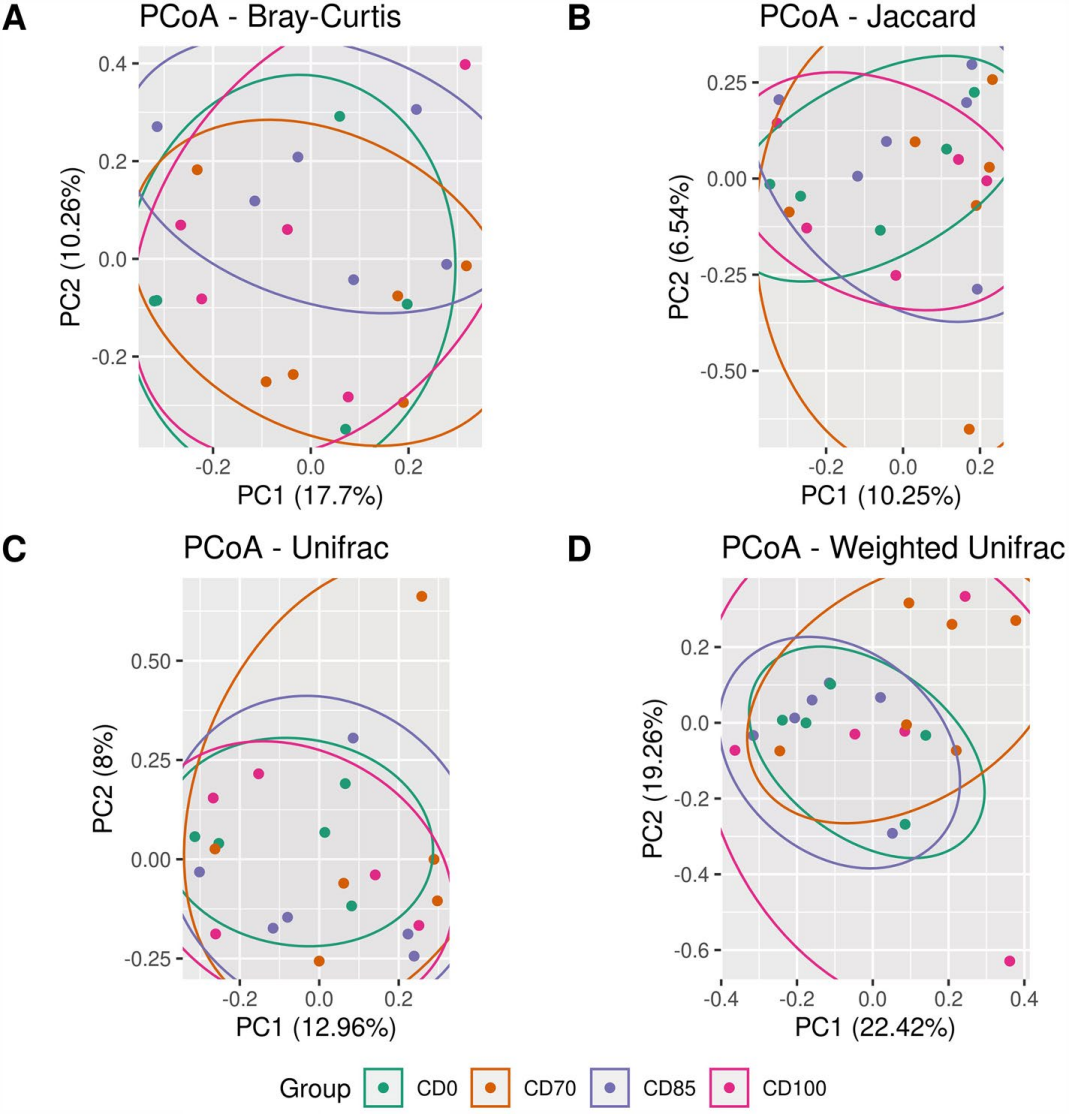
Beta diversity estimated by parameters Bray–Curtis (**A**), Jaccard (**B**), Unifrac (**C**), and Weighted Unifrac (**D**) in grower pigs (n = 6 per treatment) fed 1 of 4 dietary treatments: a control diet containing isolated phytase and xylanase valued at 40 kcal of ME/kg (CD0), CD0 + β-mannanase (0.3 g/kg valued at 30 kcal of ME/kg) (CD70), CD0 + β-mannanase (0.3 g/kg valued at 45 kcal of ME/kg) (CD85), and CD0 + β-mannanase (0.3 g/kg valued at 60 kcal of ME/kg) (CD100).

There was no effect of dietary treatment on the Chao1, observed OTUs, and Fisher indices (Fig. 2A, B, and C, respectively). Interestingly, the results of alpha diversity analysis indicated a difference (*P* < 0.05) in Simpson's, Shannon, and Pielou indices in pigs fed CD85 diet compared to animals consuming CD100 diet (Fig. 2D, E, and F, respectively). In addition, no effect was observed on beta diversity estimated by parameters Bray–Curtis, Jaccard, and Unifrac (Fig. 3A, B, and C, respectively); however, there was a trend (*P* = 0.068) in the beta diversity analysis by the Weighted Unifrac parameter (Fig. 3D).

**Figure 4:**
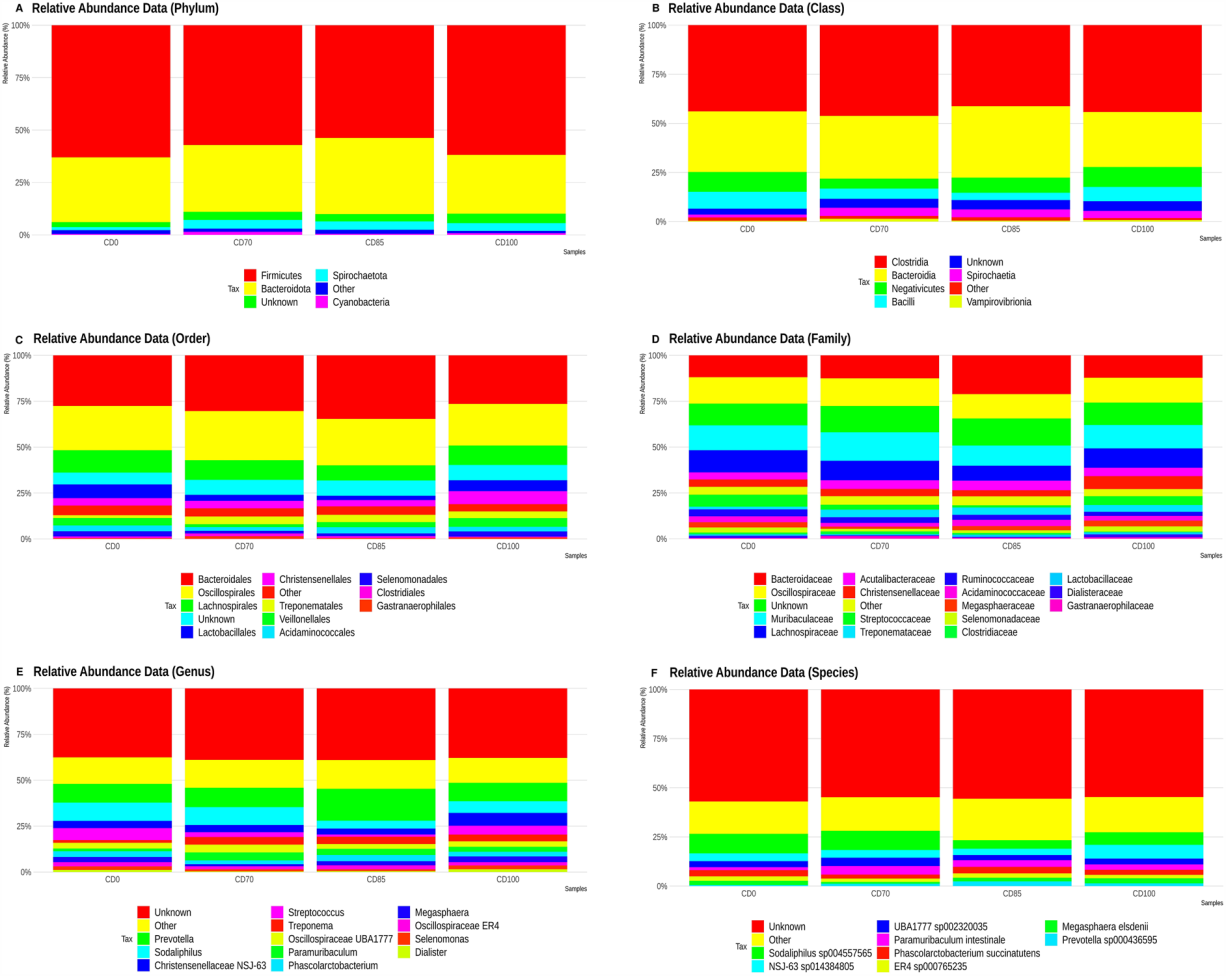
Relative abundance of phyla (**A**), classes (**B**), orders (**C**), families (**D**), genera (**E**), and species (**F**) presents in grower pigs (n = 6 per treatment) fed 1 of 4 dietary treatments: a control diet containing isolated phytase and xylanase valued at 40 kcal of ME/kg (CD0), CD0 + β-mannanase (0.3 g/kg valued at 30 kcal of ME/kg) (CD70), CD0 + β-mannanase (0.3 g/kg valued at 45 kcal of ME/kg) (CD85), and CD0 + β-mannanase (0.3 g/kg valued at 60 kcal of ME/kg) (CD100).

In the present study, the most abundant phyla were Firmicutes, Bacteroidota (previously described as Bacteroidetes), Spirochaetota (previously described as Spirochaetes), and Cyanobacteria (Fig. 4A). In addition, the classes Clostridia, Bacteroidia, Negativicutes, Bacilli, Spirochaetia, and Vampirovibrionia showed the highest abundances (Fig. 4B). The most abundant orders were Bacteroidales, Oscillospirales, Lachnospirales, Lactobacillales, Christensenellales, Treponematales, Veillonellales, Acidaminococcales, Selenomonadales, Clostridiales, and Gastranaerophilales (Fig. 4C). The families Bacteroidaceae, Oscillospiraceae, Muribaculaceae, Lachnospiraceae, Acutalibacteraceae, Christensenellaceae, Streptococcaceae, Treponemataceae, Ruminococcaceae, Acidaminococcaceae, Megasphaeraceae, Selenomonadaceae, Clostridiaceae, Lactobacillaceae, Dialisteraceae, and Gastranaerophilaceae had relative abundance (Fig. 4D). Genera that had relative abundance were *Prevotella*, *Sodaliphilus*, *Christensenellaceae NSJ-63*, *Streptococcus*, *Treponema*, *Oscillospiraceae UBA1777*, *Paramuribaculum*, *Phascolarctobacterium*, *Megasphaera*, *Oscillospiraceae ER4*, *Selenomonas*, and *Dialister* (Fig. 4E). Species that showed relative abundance were *Sodaliphilus sp004557565*, *NSJ-63 sp014384805*, *UBA1777 sp002320035*, *Paramuribaculum intestinale*, *Phascolarctobacterium succinatutens*, *ER4 sp000765235*, *Megasphaera elsdenii* and *Prevotella sp000436595* (Fig. 4F).

**Figure 5:**
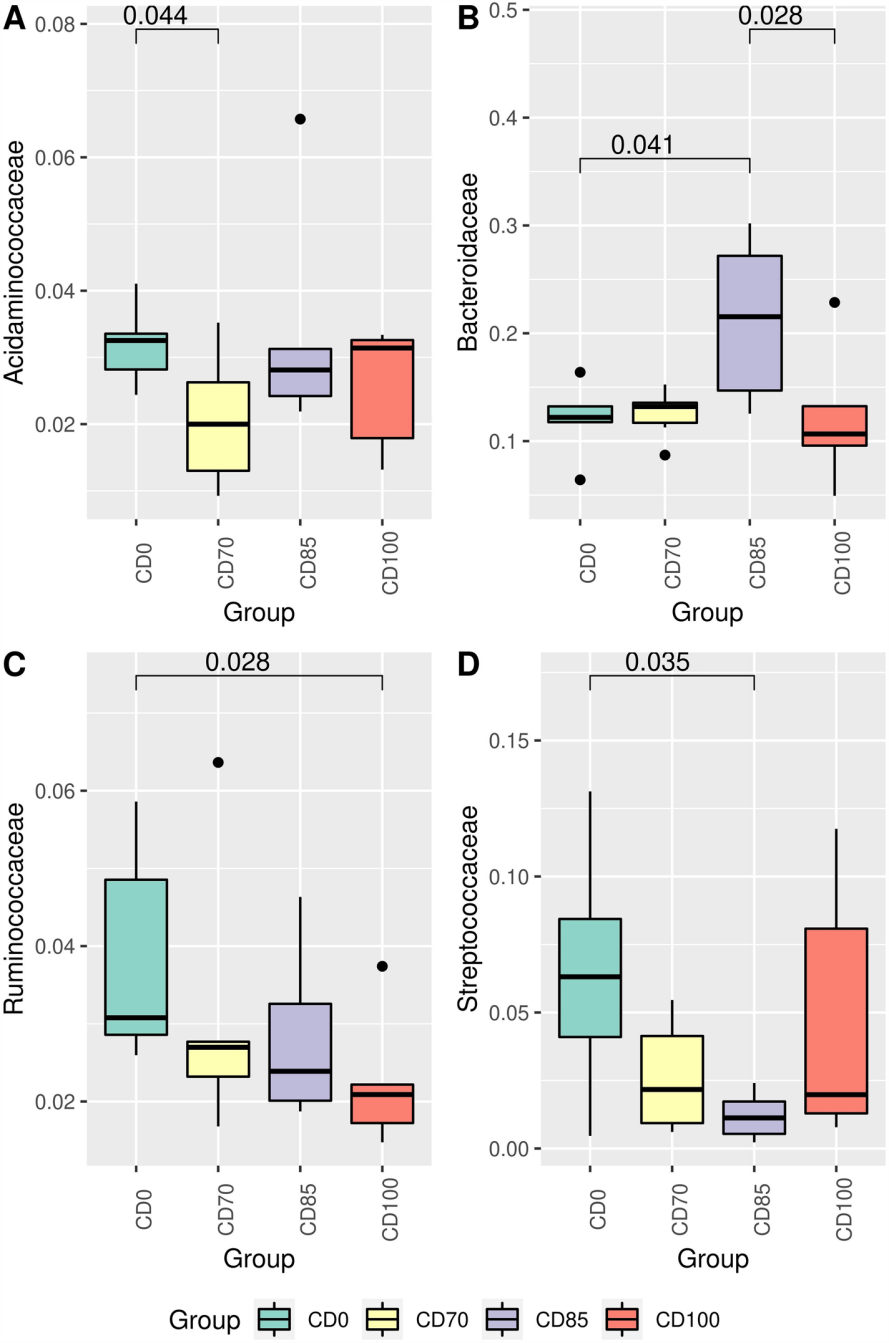
Relative abundance of Acidaminococcaceae (**A**), Bacteroidaceae (**B**), Ruminococcaceae (**C**) and Streptococcaceae (**D**) in grower pigs (n = 6 per treatment) fed 1 of 4 dietary treatments: a control diet containing isolated phytase and xylanase valued at 40 kcal of ME/kg (CD0), CD0 + β-mannanase (0.3 g/kg valued at 30 kcal of ME/kg) (CD70), CD0 + β-mannanase (0.3 g/kg valued at 45 kcal of ME/kg) (CD85), and CD0 + β-mannanase (0.3 g/kg valued at 60 kcal of ME/kg) (CD100). Means differed by Wilcoxon test (*P* < 0.05).

**Figure 6:**
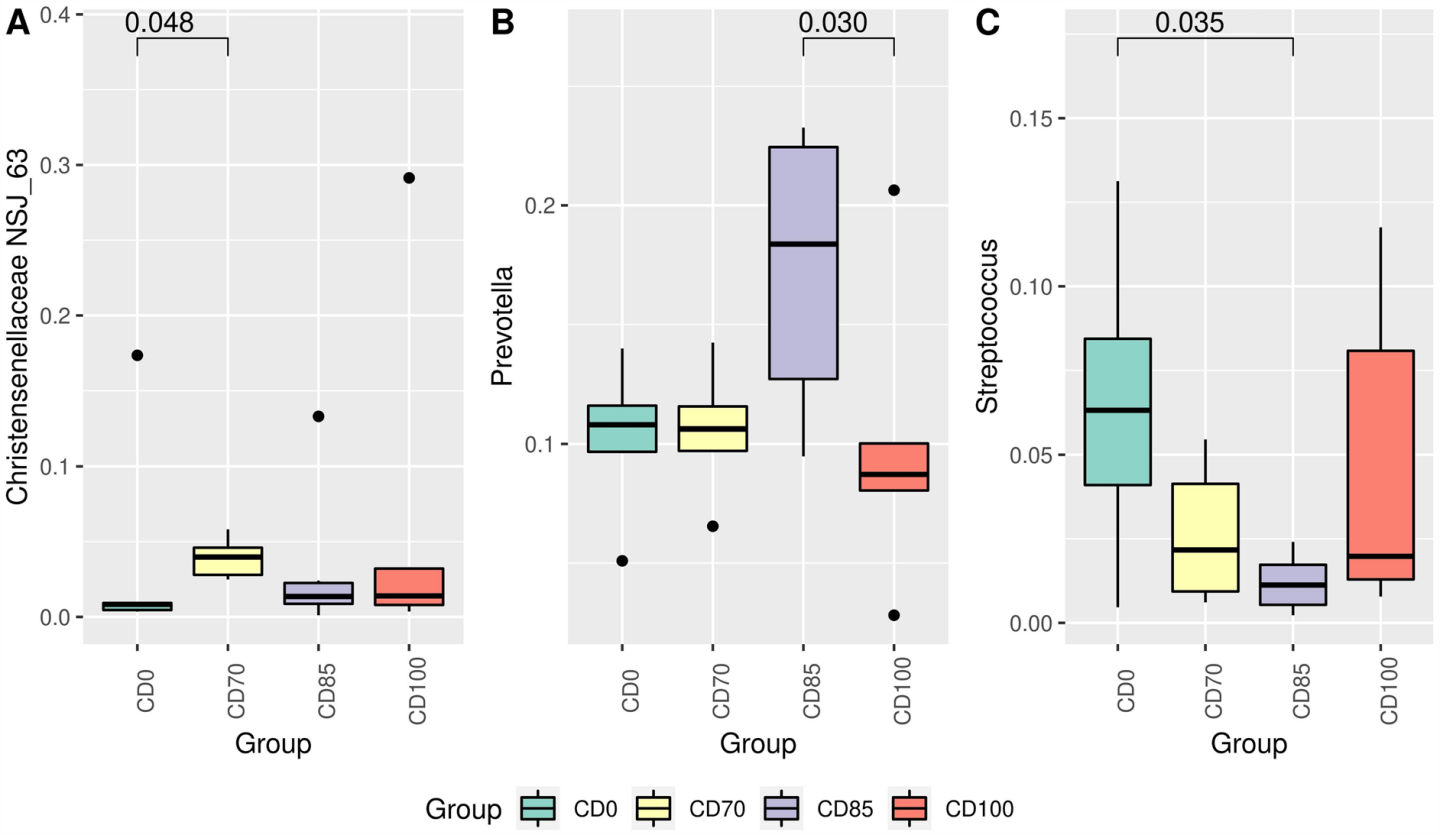
Relative abundance of *Christensenellaceae NSJ-63* (**A**), *Prevotella* (**B**), and *Streptococcus* (**C**) in grower pigs (n = 6 per treatment) fed 1 of 4 dietary treatments: a control diet containing isolated phytase and xylanase valued at 40 kcal of ME/kg (CD0), CD0 + β-mannanase (0.3 g/kg valued at 30 kcal of ME/kg) (CD70), CD0 + β-mannanase (0.3 g/kg valued at 45 kcal of ME/kg) (CD85), and CD0 + β-mannanase (0.3 g/kg valued at 60 kcal of ME/kg) (CD100). Means differed by Wilcoxon test (*P* < 0.05).

The Acidaminococcaceae family was more abundant (*P* < 0.05) in pigs fed CD0 compared to animals fed CD70 (Fig. 5A). In addition, animals that consumed the CD85 diet showed (*P* < 0.05) higher abundance in the Bacteroidaceae family than those fed CD0 or CD100 diets (Fig. 5B). The family Ruminococcaceae was more abundant (*P* < 0.05) in pigs fed CD0 diet compared to animals that received the CD100 diet (Fig. 5C). However, animals that consumed the CD0 diet showed (*P* < 0.05) greater abundance in the Streptococcaceae family (Fig. 5D), and in the genus *Streptococcus* than those fed CD85 diet (Fig. 6C).

**Figure 7:**
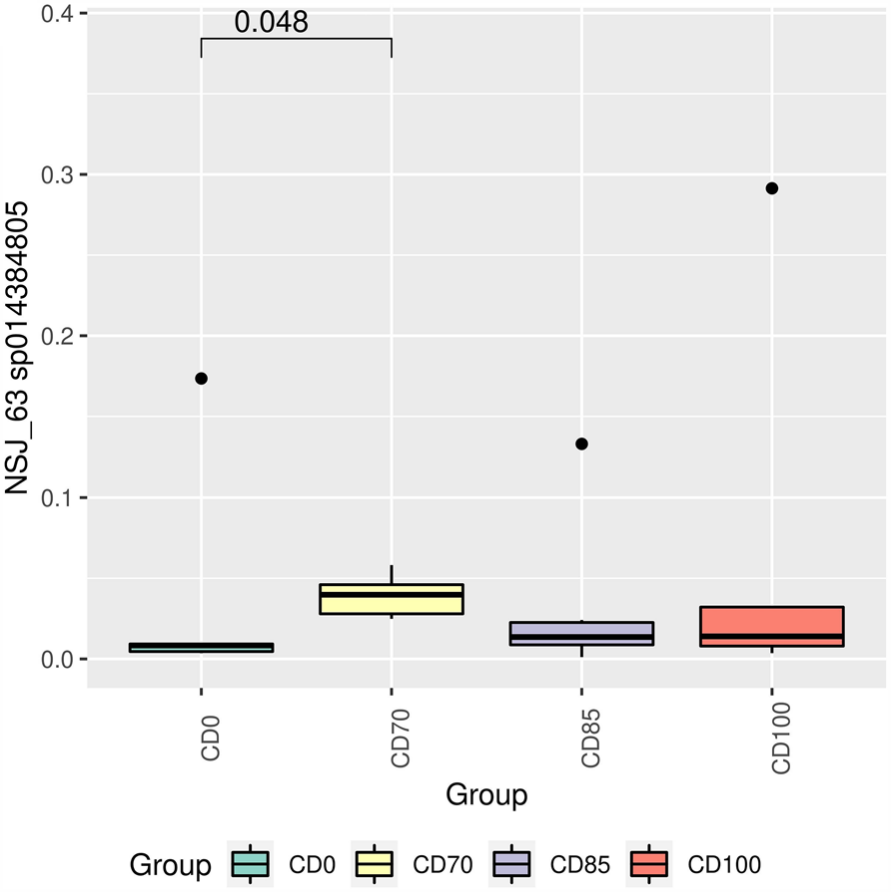
Relative abundance of *NSJ-63 sp014384805* in grower pigs (n = 6 per treatment) fed 1 of 4 dietary treatments: a control diet containing isolated phytase and xylanase valued at 40 kcal of ME/kg (CD0), CD0 + β-mannanase (0.3 g/kg valued at 30 kcal of ME/kg) (CD70), CD0 + β-mannanase (0.3 g/kg valued at 45 kcal of ME/kg) (CD85), and CD0 + β-mannanase (0.3 g/kg valued at 60 kcal of ME/kg) (CD100). Means differed by Wilcoxon test (*P* < 0.05).

**Figure 8:**
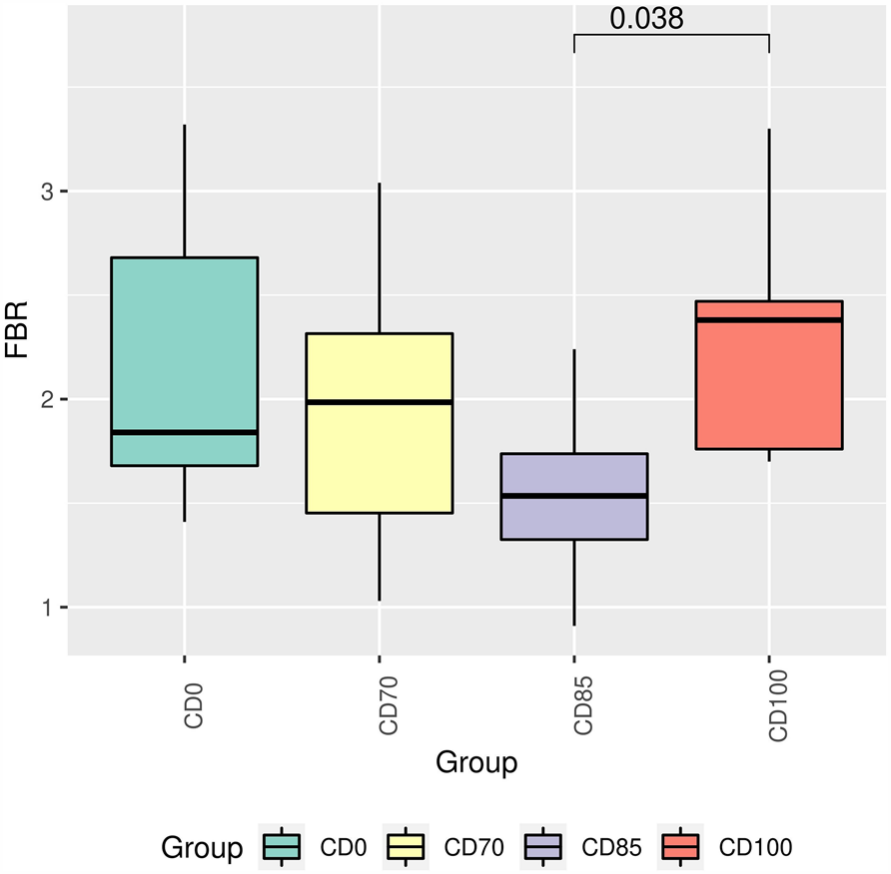
Firmicutes:Bacteroidetes ratio (FBR) in grower pigs (n = 6 per treatment) fed 1 of 4 dietary treatments: a control diet containing isolated phytase and xylanase valued at 40 kcal of ME/kg (CD0), CD0 + β-mannanase (0.3 g/kg valued at 30 kcal of ME/kg) (CD70), CD0 + β-mannanase (0.3 g/kg valued at 45 kcal of ME/kg) (CD85), and CD0 + β-mannanase (0.3 g/kg valued at 60 kcal of ME/kg) (CD100). Means differed by Wilcoxon test (*P* < 0.05).

The genus *Christensenellaceae NSJ-63* (Fig. 6A) and the species *NSJ-63 sp014384805* (Fig. 7) were more abundant (*P* < 0.05) in pigs that received the CD70 diet when compared to animals fed CD0 diet. In addition, pigs that consumed the CD85 diet exhibited (*P* < 0.05) higher abundance of the genus *Prevotella* than animals fed CD100 diet (Fig. 6B). Pigs fed CD100 diet exhibited (*P* < 0.05) higher Firmicutes:Bacteroidota ratio (FBR) compared to animals that received the CD85 diet (Fig. 8).

## Discussion

In the present study, the animals remained healthy throughout the experimental period. Overall, animals fed reduced ME-reduced diets supplemented with β-mannanase showed no impairment in growth performance compared to animals that consumed the CD0 diet. Previously, a 61 kcal ME/kg reduction in the diet supplemented with 0.01% xylanase affected the ADFI and G:F in grower pigs, but no influence was observed with 25 kcal ME/kg and 24 kcal ME/kg reductions in the diet of finisher pigs^13^. In addition, diets with reduced 120 kcal ME/kg when supplemented with 0.05% mannanase alone negatively affected the performance of grower pigs^14^.

Although the present study evidenced a loss of response on ultra-sounded backfat thickness and loin depth, there is a gap between different studies for this variable^13,14^, because a gradual utilization of dietary energy promoted by exogenous enzyme supplementation may occur in pigs fed diets without ME reduction^13^. This result suggested that the extra energy provided by the enzymes tested in the CD0 diet did not promote fat deposition or change loin depth in grower pigs, as observed by Cho et al.^14^ and Silva et al.^6^.

In the present study, regarding the non-starch polysaccharides (NSP) content present in corn grain (7.86% CP) and soybean meal (45.4% CP), the experimental diets as based on natural matter were composed of 6.8% to 16.4% NSP, respectively^1^. The combined effect of the enzymes tested promoted higher ATTD of nutrients and digestible energy, because a degradation of NSP occurs to provide extra energy to the animal^13^. In addition, hydrolysis of structural carbohydrates into smaller units results in additional energy for pig metabolism when fed diets containing β-mannanase^7^ and xylanase^9^. The effects of phytase on phytate hydrolysis and availability of amino acids that can provide energy must also be taken into consideration^6^; however, this improvement in amino acid digestibility has been inconsistent^15^.

The growth performance of the animals was not compromised and, hence, confirmed the benefits in energy saving when diets are supplemented with β-mannanase due to the increased activity of host enzymes^7^. This enzyme has the role of reducing energy expenditure by breaking down β-mannans present in plant-based ingredients that promote unnecessary immune activation, as reported by Vangroenweghe et al.^3^. This fact was reflected in greater nutrient and energy ATTD coefficients in pigs fed CD85 or CD100 diets because the enzymes tested also reduce the viscosity of the digest by the breakdown of NSP^13^, increase the availability of nutrients, improve the integrity and functionality of the intestinal mucosa, and minimize the presence of undesirable fermentable substrates in the distal small intestine^5,8,11^. As a result, there is greater nutrient absorption by enterocytes in the small intestine of pigs^2^.

In the current study, a reduction of 70 kcal of ME/kg in diets supplemented with β-mannanase containing xylanase-phytase did not promote greater ATTD in pigs, but daily feed intake was similar among animals as an attempt to compensate for the lower nutrient ATTD. Such a result did not negatively affect growth performance, nor in intestinal disorders such as altered fecal score and digesta passage rate. This would compromise nutrient usage due to less contact between enzyme and metabolizable substrate^11^. This finding indicated that the effects of ATTD by the enzymes tested are directly affected by the ME content in the diets, in agreement with those reported by Cho et al.^14^.

Regarding the blood biochemical profile, although a trend was clear in triglyceride concentration as ME content was reduced probably due to the levels of soybean oil in the diets, we observed no effect of dietary treatments. In addition, the improved nutrient and energy ATTD did not reflect in alterations in the blood biochemical profile, contrary to what was reported by Cho and Kim^13^, who observed an increased glucose concentration in grower pigs fed a diet reduced by 120 kcal ME/kg containing a 0.025% mannanase and 0.025% xylanase complex. The aforementioned authors attributed this result to the fact that the β-mannanase-xylanase enzyme successfully hydrolyzed NSP. A previous study reported that β-mannans interfere with glycemic metabolism by altering blood glucose and insulin concentrations^16^, a fact not observed in the present study.

Notably, animals fed CD100 diet showed a reduction in serum IL-1β concentration compared to animals fed the diet without β-mannanase supplementation. This fact is attributed to the potential of β-mannanase to regulate the inflammatory response^7^ that may exert negative effects on the health of animals. Our result corroborates the report of Kiarie et al.^8^, who observed lower serum IL-1α concentration in young pigs fed β-mannanase-based diets. The authors related this result to the prevention of β-mannanase in an immune response demanding unnecessary energy expenditure, because there is an induction of pro-inflammatory cytokines such as IL-1β by NSP via macrophage stimulation^17^.

In our study, supplementation of β-mannanase in diets strongly suppressed the production of the cytokine IL-10, but no alterations on IL-4, IL-6, IL-8, IL-12/23p40, IFN-alpha, IFN-gamma, and TNF-alpha. These different results suggest that β-mannanase in diet causes a dominance state in T helper 2 (Th2) cells due to its ability to reduce the allergenic effect of β-mannans, promoting a change in the immune response. However, such findings need to be interpreted with caution because IL-1β acts as an inflammatory mediator that is suppressed by Th2 cells via promotion of IL-10 production^18^, differing to the results found in pigs fed CD0 diet.

The bacterial diversity present in the gastrointestinal tract plays a role in modulating intestinal functionality and is essential for metabolism and ATTD of nutrients^4,5^. In general, the balance of the commensal microbiota is fundamental to the health of the host (e.g. pigs) because it is linked to the diversity of genera and species, which reduce and protect against pathogen invasion, as well as synthesize antimicrobial substances^19^. In addition, the phyla Firmicutes and Bacteroidota were the most abundant in the intestinal microbiome in pigs with importance for maintaining gastrointestinal homeostasis^20^. This is in the same line of results as Kim et al.^21^ and Gresse et al.^22^.

The results indicated that β-mannanase supplementation in ME-reduced diets promoted striking changes in the abundance of microbial populations, demonstrating an ability to favor intestinal microbial ecology, as reported by Kiarie et al.^8^, who stated a beneficial modulation in the intestinal microbiota due to the positive effects of the β-mannanase enzyme (e.g. reduction of pathogenic bacteria and intestinal inflammation). This was observed in increased measures of alpha diversity in pigs fed CD85 diet compared to animals fed CD100 diet, and a trend was observed in beta diversity. These differences in microbial diversity could be supported by the higher ATTD of nutrients and substrates available exclusively to the microbiota in the large intestine^11^. However, in the current study, reduced ME in the pig diets may have impacted the alpha diversity, regardless of β-mannanase supplementation, since there was no difference between animals fed CD0 and CD85 diets. We hypothesized that the reduction of 100 kcal ME/kg diet promoted changes in the composition of the fecal microbiome because this can alter the availability and composition of substrates for microbial fermentation. Although animals receiving the CD85 or CD100 diets had the highest ATTD coefficients, the release of sugars and nitrogen from the plant ingredients by the action of the β-mannanase-xylanase enzymes^8^ may have been intended for purposes other than for the intestinal microbiota of pigs fed CD100 diet.

We evidenced that pigs fed the reduced diet of 100 kcal ME/kg showed a lower abundance of microorganisms of the Ruminococcaceae family, responsible for the production of xylanases, cellulases, α-glucosidases, α- and β-galactosidases for enhanced energy utilization^23^. In addition, these microorganisms degrade and metabolize complex plant carbohydrates^24^. When the diet's ME is reduced, it can alter the availability of fermentable carbohydrates and fiber substrates. Consequently, this may affect the growth and activity of Ruminococcaceae family, potentially leading to changes in their abundance or functional roles within the intestinal microbiome.

Abundance of the Acidaminococcaceae family in pigs is associated with increased short-chain fatty acids production, as evidenced by Zhang et al.^25^. However, some members of this family are considered undesirable in the composition of the intestinal microbiota, but little is known about the specific role and function of the Acidaminococcaceae family^26^, making it difficult to interpret our result as a beneficial effect. Reduced ME content in the diet can result in decreased availability of amino acids, because amino acids are used as an energy source in inherent cases (e.g. reduced energy content). This change in nutrient availability may influence the relative abundance of members of the Acidaminococcaceae family, because it is involved in the metabolic processes of amino acid fermentation^27^, potentially leading to shifts in their population size. In the present study, all diets were isoaminoacidic and the difference observed was between animals fed CD0 compared to CD70 diets. Hence, additional investigations are needed to better understand the effects of the enzymes tested in ME-reduced diets on the Acidaminococcaceae family of grower pigs.

The greater relative abundance of the Streptococcaceae family and *Streptococcus* genus in pigs that had the CD0 diet than animals fed CD85 diet suggested compromised intestinal health^28^ because these microorganisms have been related to inflammatory processes at the intestinal level^29^. Interestingly, pigs that received the CD85 diet showed an increase in the relative abundance of the Bacteroidaceae family. The abundance of this family was associated with high-protein diets but lower fermentable fiber content in the human intestinal microbiota^30^. When analyzed together, the higher relative abundance of the Ruminococcaceae family in pigs that received the CD0 diet contributed to the lower abundance of the Bacteroidaceae family, because bacteria of the Bacteroidaceae family do not tolerate acidic pH conditions due to the fermentative activity of bacteria represented by the Ruminococcaceae family^31^.

The genus *Prevotella* was evidenced to be more abundant in pigs fed CD85 than those fed CD100. This result indicated that these differences are supported by the reduction in energy content in the diet, because *Prevotella* plays a role in carbohydrate metabolism, such as polysaccharide degradation and oligosaccharide utilization^32,33^. In our study, it is plausible that a decrease in dietary ME affects the growth and survival of certain microbial taxa. Some microorganisms may be more dependent on specific substrates or energy sources, and their abundance may reduce as energy availability decreases. On the other hand, certain microbial populations that can adapt to low energy diets or utilize alternative energy sources can thrive, causing an altered microbial community structure.

To date, no study has reported the abundance of the *Christensenellaceae NSJ-63* genus and *NSJ-63 sp014384805* species in the fecal microbiome in pigs, as observed in our study in animals consuming the CD70 diet compared to pigs fed CD0 diet. However, the abundance of the Christensenellaceae family has been related to higher feed efficiency rate in pigs^34^, health status^35^ and ability to produce short-chain fatty acids as end products of sugar fermentation^36^. Although no significant effect was detected on the Christensenellaceae family, the greater abundance of the genus and species in animals fed CD70 diet explains the lack of impairment on growth performance.

The FBR is accepted as a beneficial evaluative variable for intestinal health and hence changes in this ratio may promote several pathologies^37^. In a previous experiment, higher FBR was related to improved energy efficiency and growth performance in pigs^38^. However, this result should be interpreted with caution in our study because pigs that received the CD100 diet had similar growth performance and nutrient ATTD as pigs that consumed the CD85 diet. In summary, it appears that supplementation of β-mannanase in ME-reduced diets containing xylanase-phytase favored the establishment of the microbiotypes identified in our study, allowing for differences in the fecal microbiome in grower pigs. Therefore, although a reduction in ME may affect alpha and beta diversity in pigs, this is only one aspect to consider when evaluating the complex interactions between diet, fecal microbiome, and animal health.

## Conclusions

Based on the criteria evaluated in the present study, the supplementation of β-mannanase in diets containing xylanase-phytase allowed to save 85 to 100 kcal of ME/kg by sustaining growth performance and improving nutrient digestibility, but a beneficial and dynamic modulation in microbial alpha diversity in pigs on 85 kcal of ME/kg. In addition, β-mannanase supplemented in ME-reduced diets containing xylanase-phytase promoted minor changes in inflammatory cytokines without negatively affecting the biological response of grower pigs.

## Materials and methods

### Ethics statement

All experimental protocols were approved by a named institutional and/or licensing committee Universidade Estadual do Oeste do Paraná (Unioeste, Brazil) (protocol no. 17/2022). All methods were carried out in accordance with relevant guidelines and regulations. All methods were reported in accordance with ARRIVE guidelines (https://arriveguidelines.org/arrive-guidelines).

### Animals, experimental design, housing and diets

A total of 40 hybrid male pigs (Landrace × Large White) weighing 26.09 ± 0.96 kg were assigned in a randomized complete block design based on body weight within 4 dietary treatments and 10 pen replicates, with one animal per pen as the experimental unit.

The animals were weighed, identified with numbered ear tags, and housed in a masonry facility with ceramic tiles, with fully compacted floor pens (6.34 m^2^), arranged in two rows, divided by a central corridor, as described by Genova et al.^39^. All pens were equipped with a semi-automatic feeder located in the front and a drinking fountain.

The ambient temperature (20.40 ± 6.65 °C) and relative humidity (63.66 ± 19.30%) were recorded during the experimental period using a datalogger with digital display (Hygro-Thermometer, model RT811) installed in the center of the facility. Control of temperature and ventilation inside the facility was performed with the aid of side curtains and trees on both sides.

The experimental period lasted 42 days and was divided into two phases: grower I (0 to 25 days) and grower II (25 to 42 days). The diets were formulated based on ground corn and soybean meal, supplemented with industrial amino acids following the nutritional requirements proposed by Rostagno et al.^1^ (Table 4). All diets were provided in the meal form, ad libitum and were isonutritional with variations only in the content of soybean oil and inert (kaolin).

**Table 4:** Composition of diets provided to grower pigs (as fed basis, %). ^a^Content per kg of premix: Mn sulfate, 5400 mg/kg; Zn oxide, 13.50 g/kg; Fe sulfate, 10.50 g/kg; Cu sulfate, 2100 mg/kg; I, 150 mg/kg; vitamin A, 900,000 IU/kg; vitamin D_3_, 180,000 IU/kg; vitamin E, 3000 IU/kg; vitamin K_3_, 270 mg/kg; vitamin B_1_, 120 mg/kg; vitamin B_2_, 570 mg/kg; vitamin B_6_, 120 mg/kg; vitamin B_12_, 2100 mcg/kg; niacin, 3000 mg/kg; pantothenic acid, 1950 mg/kg; folic acid, 75 mg/kg; Se, 90 mg/kg; phytase, 166.66 U/g; xylanase, 333.33 U/g. ^b^Standardized ileal digestible. ^c^Standardized total tract digestible.

Item	Grower I					Grower II			
	CD0	CD70	CD85	CD100		CD0	CD70	CD85	CD100
Ingredients (%)									
Ground corn, 7.86% CP	70.36	70.36	70.36	70.36		72.71	72.71	72.71	72.71
Soybean meal, 45.4% CP	24.75	24.75	24.75	24.75		22.20	22.20	22.20	22.20
Dicalcium phosphate	1.68	1.68	1.68	1.68		1.41	1.41	1.41	1.41
Calcitic limestone	0.68	0.68	0.68	0.68		0.59	0.59	0.59	0.59
Inert (kaolin)	–	0.33	0.51	0.69		–	0.33	0.51	0.69
Soybean oil	0.75	0.39	0.21	0.03		1.44	1.07	0.89	0.71
Common salt	0.44	0.44	0.44	0.44		0.41	0.41	0.41	0.41
Premix^a^	0.30	0.30	0.30	0.30		0.30	0.30	0.30	0.30
Lysine sulfate, 54.6%	0.56	0.56	0.56	0.56		0.55	0.55	0.55	0.55
DL-methionine, 99.5%	0.16	0.16	0.16	0.16		0.14	0.14	0.14	0.14
L-threonine, 96.8%	0.17	0.17	0.17	0.17		0.16	0.16	0.16	0.16
L-tryptophan, 99%	0.04	0.04	0.04	0.04		0.03	0.03	0.03	0.03
L-valine, 95.5%	0.06	0.06	0.06	0.06		0.01	0.01	0.01	0.01
β-mannanase	-	0.03	0.03	0.03		-	0.03	0.03	0.03
Enramycin	0.006	0.006	0.006	0.006		0.006	0.006	0.006	0.006
Calculated chemical composition									
Metabolizable energy, kcal/kg	3.260	3.230	3.215	3.200		3.310	3.280	3.265	3.250
Crude protein, %	17.50	17.50	17.50	17.50		16.44	16.44	16.44	16.44
SID^b^ lysine, %	1.10	1.10	1.10	1.10		1.03	1.03	1.03	1.03
SID methionine + cysteine, %	0.65	0.65	0.65	0.65		0.61	0.61	0.61	0.61
SID threonine, %	0.71	0.71	0.71	0.71		0.67	0.67	0.67	0.67
SID tryptophan, %	0.23	0.23	0.23	0.23		0.21	0.21	0.21	0.21
SID valine, %	0.80	0.80	0.80	0.80		0.71	0.71	0.71	0.71
Total calcium, %	0.77	0.77	0.77	0.77		0.66	0.66	0.66	0.66
STTD^c^ phosphorus, %	0.38	0.38	0.38	0.38		0.33	0.33	0.33	0.33
Total sodium, %	0.19	0.19	0.19	0.19		0.18	0.18	0.18	0.18

The dietary treatments were composed of: 1) a control diet containing isolated phytase and xylanase valued at 40 kcal of ME/kg (CD0), 2) CD0 + β-mannanase (0.3 g/kg valued at 30 kcal of ME/kg) (CD70), 3) CD0 + β-mannanase (0.3 g/kg valued at 45 kcal of ME/kg) (CD85), and 4) CD0 + β-mannanase (0.3 g/kg valued at 60 kcal of ME/kg) (CD100). The ME valorization in the diets was based on the requirement of 3,300 kcal ME/kg and 3,350 kcal ME/kg for pigs in grower I and II phases, respectively^1^.

### Traits of the tested enzymes

Xylanase (Sunhy Biology Co., Ltd, Wuhan, HB, China; registration no. PR-08978 03,462) was a product obtained from *Trichoderma longibrachiatum* with the activity of 10,000 U/g*.* A U of xylanase is the amount of enzyme that releases 1 micromol of reducing sugar from a xylan solution (5 mg/mL) at 37 °C and pH 5.5. Phytase (Sunhy Biology Co., Ltd, Wuhan, HB, China; registration no. PR 000,267–4.000005) was a product from *Aspergillus niger* with the activity of 1,000 U/g of dry solid at 37 °C and pH 5.5. β-mannanase (Elanco Animal Health, Inc., São Paulo, SP, Brazil; registration no. SP-59122 30,011, Hemicell™ HT) was obtained from *Paenibacillus lentus* with the activity of 160,000 U/g*.* A U of β-mannanase is the amount of enzyme that releases 0.72 mcg of reducing sugars (equivalent to D-mannose) per min from goma locust (mannans concentration of 88%) at 40 °C and pH 7.5.

### Growth performance

Diet and water were provided ad libitum to the animals. Diets were weighed daily before feeding and leftovers and waste were manually collected for determination of the average daily feed intake (ADFI, kg/day). Animals were weighed on days 0, 25, and 42 to monitor initial body weight (IBW, kg), final body weight (FBW, kg), daily weight gain (DWG, kg/day), and calculate gain to feed ratio (G:F, kg:kg).

### Fecal score

The assessment was performed through partial collection of feces at the end of the growth phases, following the procedures for determining fecal score^39^. Prior to the beginning of collection, the pens were cleaned (0800) and the animals were monitored for 12 h uninterrupted. During this period, fecal samples were collected several times immediately after the animals defecated, except for the fraction of feces that was in contact with the floor of the pens. Immediately, the sampled material was stored in the identified polyethylene plastic bags and placed in thermal boxes (4ºC) until the end of the collection period. Subsequently, the feces corresponding to each pen were homogenized, an aliquot was taken in duplicate (110 g each), weighed on a scale (bel engineering, model M4102, Monza, LOM, Italy) and dried in a forced ventilation oven (Tecnalbrand, SF-325 NM model; Piracicaba, SP, Brazil) at 55 °C for 72 h to determine dry matter^40^. The values obtained were classified according to fecal consistency, following the adapted methodology^41^.

### Ultra-sounded backfat thickness and loin depth

In vivo loin depth and backfat thickness were assessed in all animals in the P2 lumbar region^42^ at the end of growth II phase (on day 42) using ultrasound equipment (Aloka Co., Ltd., SSD-500 vet, Tokyo, Japan) equipped with a 15-cm linear transducer and 3.5-MHz frequency operation. An amount (≅ 45 mL) of vegetable oil was used as the acoustic couplant after the region was trichotomized using a painless epilator device^39^. Animals were kept individually in metal cages for data recording immediately after capturing the lumbar region.

### Sampling, preparation and analysis of blood metabolites

The procedures adopted agree with those described in a previous study^39^. All animals fasted for 8 h at the end of the grower II phase (on day 42). Blood collection (≅ 10 mL) was performed via puncture of the anterior cranial vena cava. Blood was collected using 20 mL syringes and 1.2 × 40 mm gauge needles, and doses were transferred to one of three tubes (glass vacuum blood collection tube, Labingá, PR, Brazil) containing potassium fluoride, EDTA, and no anticoagulant. The tubes were previously identified, transferred into a thermal box containing ice (4 °C) and sent to the blood laboratory for further analysis. Then, the tubes containing the samples were centrifuged (Centrilab analog centrifuge, model 80-2B, Rio de Janeiro, RJ, Brazil) at 3,000 g for 10 min. Then, ≅ 3 mL of plasma or serum were transferred to previously identified eppendorf polyethylene tubes and frozen (-18 °C) for analysis of urea (enzymatic-colorimetric method), glucose (enzymatic-colorimetric method), cholesterol (enzymatic-colorimetric method), total protein (enzymatic-biuret method), and albumin (colorimetric—bromocresol green). The analyses were performed by spectrophotometry with the aid of an analyzer (Bel brand, SPECTRO S05 model, Rio de Janeiro, RJ, Brazil), using specific ANALISA kits (Gold Analisa Diagnostic, Belo Horizonte, MG, Brazil) in the blood laboratory of Unioeste. The globulin value was calculated considering the difference between total protein and plasma albumin.

Blood samples from 8 animals per treatment were stored at -80ºC and sent to IMUNOVA laboratory (Curitiba, PR, Brazil) for simultaneous detection of IL-1β, IL-4, IL-6, IL-8, IL-10, IL-12/23p40, IFN-alpha, IFN-gamma and TNF-alpha in serum, using multiplex assay with commercial kit (Invitrogen EPX090-60,829–901) and applying the Luminex xMAP® technology, which uses magnetic nanospheres conjugated to specific antibodies for each detectable target.

### Apparent total tract digestibility of nutrients and digestible energy

The indicator insoluble acid ash (IAA, celite®) was added in the dietary treatments (10 g/kg diet) at the end of growth II phase (on day 42) to assess ATTD by partial collection of feces^44^. The diets containing the indicator were homogenized for 10 min using a vertical mixer, as performed previously^39^. Then, these diets were fed for three days before the beginning of collection. On the fourth day, partial collection of feces was performed, according to adapted methodology^44^. The start and end of diet feeding, as well as feed intake per pen were recorded. Feces were collected for 12 h on the fourth day. During collection, feces were stored in previously identified polyethylene plastic bags and kept in thermal boxes containing ice (4ºC). After the end of collection, the samples were stored in a freezer (-18 °C) for further analysis.

Subsequently, the samples were thawed, homogenized, and an aliquot was taken in technical duplicate (110 g each), weighed on a scale (bel engineering, model M4102, Monza, LOM, Italy), and dried (55 °C for a period of 72 h) in a forced ventilation oven (Tecnalbrand, SF-325 NM model; Piracicaba, SP, Brazil)^40^. Then, the samples were ground in a micro pulverizer mill (R-TE-350; Tecnal Equipment Scientific, Piracicaba, SP, Brazil), and stored in previously identified plastic containers.

The analysis of IAA was performed by digestion with hydrochloric acid (4N)^43^. The chemical composition of the diets and feces were performed according to the methodology described by Silva and Queiroz^40^ for CP by the Kjeldahl procedure, DM, mineral matter (MM), and calculation of OM. The gross energy (GE) analysis of diets and feces were performed by samples combustion in a calorimetric bomb (IKA®, model C200, Wilmington, NC, USA).

Based on the raw results, the percent IAA recovery and the ATTD coefficients of DM, CP, OM, and GE were calculated^43^. Digestible nutrient and energy values were calculated in percent of ATTD of DM, CP, OM, and kcal/kg of DE, according to the equations established by Sakomura and Rostagno^43^.

### Digesta passage rate

The assessment of the total digesta passage rate was performed at the end of the growth phases (on days 25 and 42) by fecal marker excretion (ferric oxide)^45^. A standard amount of diet was weighed adding 1.5% marker and homogenized to ensure intake in a single meal, as adopted in a previous study conducted by our team^39^. All diet present in the pen feeders was removed 1 h prior to the start of the marker diet and stored in labeled containers to be returned to the feeder. The feeding of the marked diets was performed manually in an orderly manner according to the time of withdrawal of the diet without the marker. The time of feeding and the time when the animals completed the total consumption of the marked diet (time 0) was recorded individually per pen. Monitoring of the pens was organized to identify the marked feces and record the respective time. The total digesta passage rate was calculated based on the time (in min) elapsed between total consumption of the marked diet and the onset of excretion of marked feces.

### Fecal microbiome

Fecal samples from the rectum (≅ 2 g) of 6 pigs per treatment were manually collected at the end of growth II phase (on day 42) and immediately transferred to sterile Eppendorf plastic tubes of 3 mL using the procedure and standards described in Genova et al.^39^. Immediately, the samples were frozen at -80 °C until analysis. A commercial kit (ZR Fecal DNA MiniPrep^®^, Zymo Research South America, Botucatu, SP, Brazil) was used to extract DNA from the samples following the manufacturer's recommended protocol. The extracted DNA was quantified by spectrophotometry at 260 nm. All samples were run by electrophoresis in 1% agarose gel to assess the integrity of the extracted DNA.

A segment of approximately 460 bases of the V3–V4 hypervariable region of the 16S ribosomal RNA gene was amplified using the universal primers described by the methodology, and the following PCR conditions: 95 °C for 3 min, 25 cycles of 95 °C for 30 s, 55 °C for 30 s, and 72 °C for 30 s, followed by a step at 72 °C for 5 min. From these amplifications, the metagenomic library was built using a commercial kit (Nextera DNA Library Preparation Kit by Illumina^®^). The amplifications were pooled and subsequently sequenced on the Illumina^®^ MiSeq™ sequencer^46^.

The reads obtained on the sequencer were analyzed in the quantitative insights into microbial ecology (QIIME 2) platform^47^, following an analysis flow for removal of low-quality sequences, filtration, chimera removal and taxonomic classification. Sequences were classified into bacterial genera by recognizing amplicon sequence variants (ASVs), that is, the homology between sequences when compared against a database. Sequences were compared by the 2019 update (SILVA 138), belonging to the SILVA ribosomal sequence database^48^.

To generate the classification of bacterial communities by ASVs identification, 25,610 reads per sample were used to normalize the data and not compare samples with different number of reads. The samples of identifiers 29,160 and 29,167 were removed due to the low number of reads (< 15,000) that were recovered after the quality filtering steps.

### Statistical procedures

Before assessing the results of the one-way analysis of covariance (ANCOVA) and variance (ANOVA), the analysis of Student's standardized residuals was examined to detect outliers (values greater than or equal to three standard deviations). The normality of errors and the homogeneity of variances between treatments for the variables were previously assessed using the Shapiro–Wilk and Levene tests, respectively. For the growth performance variables, the statistical model used was: Y_ijk_ = µ + T_i_ + b_j_ + β (X_ijk_ -${\overline{\mathrm{X}} }_{\dots }$) + ε_ijk_, in which Y_ijk_ = mean observation of the dependent variable in each plot, measured in the i-th treatment class, in the j-th block and in the k-th replication; µ = effect of the overall mean; T_i_ = fixed treatment effect, for i = (1, 2, 3 and 4); b_j_ = random effect of block, for j = (1 and 2); β = regression coefficient of Y on X; X_ijk_ = mean observation of the initial body weight covariate in each plot, measured in the i-th treatment class, in the j-th block and in the k-th replication; ${\overline{\mathrm{X}} }_{\dots }$= overall mean for the covariate X; ε_ijk_ = random error associated with observation Y_ijk_. For the other variables, the statistical model used was the one mentioned above, without including the covariate effect.

The effects of treatment on the dependent variables were examined using ANCOVA or ANOVA. Multiple comparisons between treatment means were performed according to Tukey's post hoc test. These statistical analyses were performed using SAS University Edition (SAS Inst. Inc., Cary, NC, USA) procedures. All data with normal distribution were presented as means with pooled standard error of the mean.

For the fecal microbiome, the statistical comparison between groups in the alpha diversity analyses and in the relative abundances of taxa among all experimental groups was performed using the nonparametric Wilcoxon test. Statistical analyses for beta diversity were performed using the permutational multivariate analysis of variance (PERMANOVA) present in the QIIME 2 pipeline, using a number of 10,000 permutations. Alpha diversity analyses were calculated by the phyloseq^49^ and microbiome^50^ libraries. For cytokine analyses data, outliers were identified via ROUT test (Q = 1%) and the normality was assessed via D'Agostino-Pearson test. Then, statistical differences were determined using the Kruskal–Wallis test followed by Dunn's post hoc. Treatment significance was stated when *P* < 0.05 and a trend when 0.05 < *P* < 0.1.

## Data Availability

The datasets used and/or analysed during the current study available from the corresponding author on reasonable request.
